# Gestational diabetes: a contributor to long-term thyroid dysfunction and disease

**DOI:** 10.3389/fendo.2025.1656498

**Published:** 2025-08-29

**Authors:** Yu-Hsiang Shih, Chia-Jung Hsieh, Shao-Jing Wang, Chien-Hsing Lu, Jenn-Jhy Tseng

**Affiliations:** ^1^ Department of Obstetrics and Gynecology, Taichung Veterans General Hospital, Taichung, Taiwan; ^2^ Department of Public Health, Chung Shan Medical University, Taichung, Taiwan; ^3^ Department of Nursing, College of Nursing, HungKuang University, Taichung, Taiwan; ^4^ Department of Post-Baccalaureate Medicine, College of Medicine, National Chung Hsing University, Taichung, Taiwan

**Keywords:** gestational diabetes mellitus, thyroid disease, thyroid function, hyperthyroidism - diagnosis, hypothyroidism

## Abstract

**Background:**

Emerging evidence links gestational diabetes mellitus (GDM) with thyroid dysfunction, but the long-term risk of clinically diagnosed thyroid diseases after GDM remains unclear.

**Objective:**

To assess the long-term risk of specific thyroid disorders in women with prior GDM compared with those without GDM.

**Methods:**

We performed a retrospective cohort study using the TriNetX U.S. Collaborative Network, including de-identified records from >80 healthcare organizations. Women aged 16–45 years with pregnancies from 2001 to 2015 were eligible; those with pre-existing hypertension, diabetes, thyroid disease, thyroid surgery, or preeclampsia/eclampsia were excluded. GDM was identified by ICD codes. The primary outcome was first diagnosis of thyroid disorders—hyperthyroidism, hypothyroidism, toxic/non-toxic goiter, thyroiditis (acute, subacute, Hashimoto’s), benign thyroid neoplasms, and thyroid cancer—after the index pregnancy. Propensity score matching (1:1) accounted for age, race, overweight/obesity, nicotine dependence, and alcohol abuse. Follow-up extended up to 20 years. Hazard ratios (HRs) were estimated using Cox models; cumulative incidence was compared with Kaplan–Meier analysis.

**Results:**

After matching, GDM was associated with higher risks of hyperthyroidism (HR 1.60, 95% CI 1.30–1.99), hypothyroidism (HR 1.33, 95% CI 1.17–1.51), thyroiditis (HR 1.55, 95% CI 1.21–2.00), Hashimoto’s thyroiditis (HR 1.37, 95% CI 1.02–1.83), toxic goiter (HR 1.70, 95% CI 1.19–2.44), and non-toxic goiter (HR 1.26, 95% CI 1.10–1.45). No association was found for benign neoplasms or thyroid cancer. Risks were greater in women aged 35–45 years and those with BMI >25.

**Conclusion:**

GDM is linked to increased long-term risk of multiple thyroid diseases, warranting extended thyroid monitoring in older and overweight/obese women.

## Introduction

Gestational diabetes mellitus (GDM) is a common metabolic disorder affecting a significant proportion of pregnant women worldwide, with a pooled global standardized prevalence of 14.0% and a regional prevalence of 7.1% (7.0–7.2%) in North America (1). It is characterized by glucose intolerance that first appears or is diagnosed during pregnancy, often resolving postpartum (2). While GDM is widely recognized for its short-term complications, including an increased risk of preeclampsia, cesarean delivery, and neonatal hypoglycemia, its long-term health implications extend beyond pregnancy (2). It is well established that women with a history of GDM are at increased risk for developing type 2 diabetes mellitus (T2DM), cardiovascular diseases, and metabolic syndrome (3). Several studies have demonstrated a short-term association between GDM and altered thyroid function, including elevated TSH levels and increased prevalence of thyroid autoantibodies during or shortly after pregnancy (4, 5). From a biochemical perspective, insulin resistance—a hallmark of GDM—may influence thyroid hormone metabolism through altered deiodinase activity, changes in leptin and adipokine signaling, and pro-inflammatory cytokine pathways (6–8).Additionally, elevated estrogen levels during pregnancy can increase thyroxine-binding globulin (TBG), altering free thyroid hormone levels (9). The autoimmune component of thyroid dysfunction may also be amplified by the systemic inflammatory environment characteristic of GDM (4), further suggesting a complex interplay between glucose metabolism and thyroid regulation.

Despite these mechanistic insights, the long-term thyroid outcomes in women with prior GDM remain poorly understood. Most existing studies are limited by small sample sizes and short follow-up durations. To address this knowledge gap, we conducted a large-scale, real-world retrospective cohort study using the TriNetX database. This platform allows for comprehensive longitudinal tracking of diverse populations across multiple healthcare organizations (10).The primary objective of this study was to evaluate whether a history of GDM is associated with an increased long-term risk of developing thyroid dysfunction or disease. This design offers a unique opportunity to explore potential long-term endocrine sequelae of GDM using robust, population-based data.

## Material and method

### Data sources

The TriNetX database is a global healthcare collaboration and clinical research platform that integrates real-time electronic medical records from a consortium of healthcare organizations. It contains de-identified health information from more than 250 million individuals across over 120 participating institutions, thereby providing a robust foundation for large-scale epidemiological investigations. This extensive dataset facilitated the comprehensive analyses conducted in our study. The platform implements rigorous preprocessing procedures to address missing data and standardizes records into a unified clinical model, ensuring consistency and comparability across diverse sources. Although patients with incomplete follow-up were not excluded, no statistical imputation techniques were employed. Instead, missingness was addressed through TriNetX’s standardized preprocessing pipeline, which incorporates data harmonization across sites and the application of natural language processing algorithms to extract relevant clinical information from unstructured text. These measures enhance the completeness and reliability of the structured electronic health records used in the present analysis (10).

Moreover, TriNetX continuously updates its datasets, enabling researchers to access the most current clinical information available from contributing institutions. The platform’s integrated analytic tools and standardized coding systems—including ICD, CPT, ATC, and RxNorm—facilitate methodological transparency and reproducibility. Importantly, TriNetX operates under a rigorous data governance framework and adheres to HIPAA and other applicable international privacy regulations, thereby ensuring ethical data stewardship while maintaining scientific rigor (11).

### Ethics statement

Because the data is anonymized, the requirement for informed consent was waived. It is essential to emphasize that TriNetX rigorously complies with the standards set by the Health Insurance Portability and Accountability Act and the General Data Protection Regulation. Moreover, the Western Institutional Review Board has approved an informed consent waiver for TriNetX, as the platform only provides aggregated counts and statistical summaries of de-identified data. Additionally, our specific use of TriNetX for this study has been approved by the Institutional Review Board (IRB) of Taichung Veterans General Hospital, under approval number CE25150B, TCVGH.

### Cohort description

This study analyzed data from January 1, 2001, to December 31, 2015, including pregnant patients aged 16 to 45 years who had no prior history of hypertension, diabetes mellitus (DM), hyperthyroidism, hypothyroidism, toxic or non-toxic thyroid goiter, acute or subacute thyroiditis, Hashimoto’s thyroiditis, iodine deficiency-related thyroid disorders, thyroid cancer, benign thyroid neoplasm, or previous thyroid surgery. In addition, patients with a history of preeclampsia or eclampsia during pregnancy were excluded.

The study population was categorized into two cohorts: one comprising patients diagnosed with gestational diabetes mellitus (GDM) during pregnancy and the other consisting of those without GDM. Although diagnostic criteria for GDM may vary slightly across regions, we primarily identified GDM cases using ICD codes available in the TriNetX database.

Although the inclusion period for eligible pregnancies spanned from 2001 to 2015, the TriNetX database provides longitudinal follow-up data through December 31, 2024. As a result, patients diagnosed earlier in the study period could be followed for up to 20 years, enabling comprehensive long-term outcome assessment.

**Figure 1:**
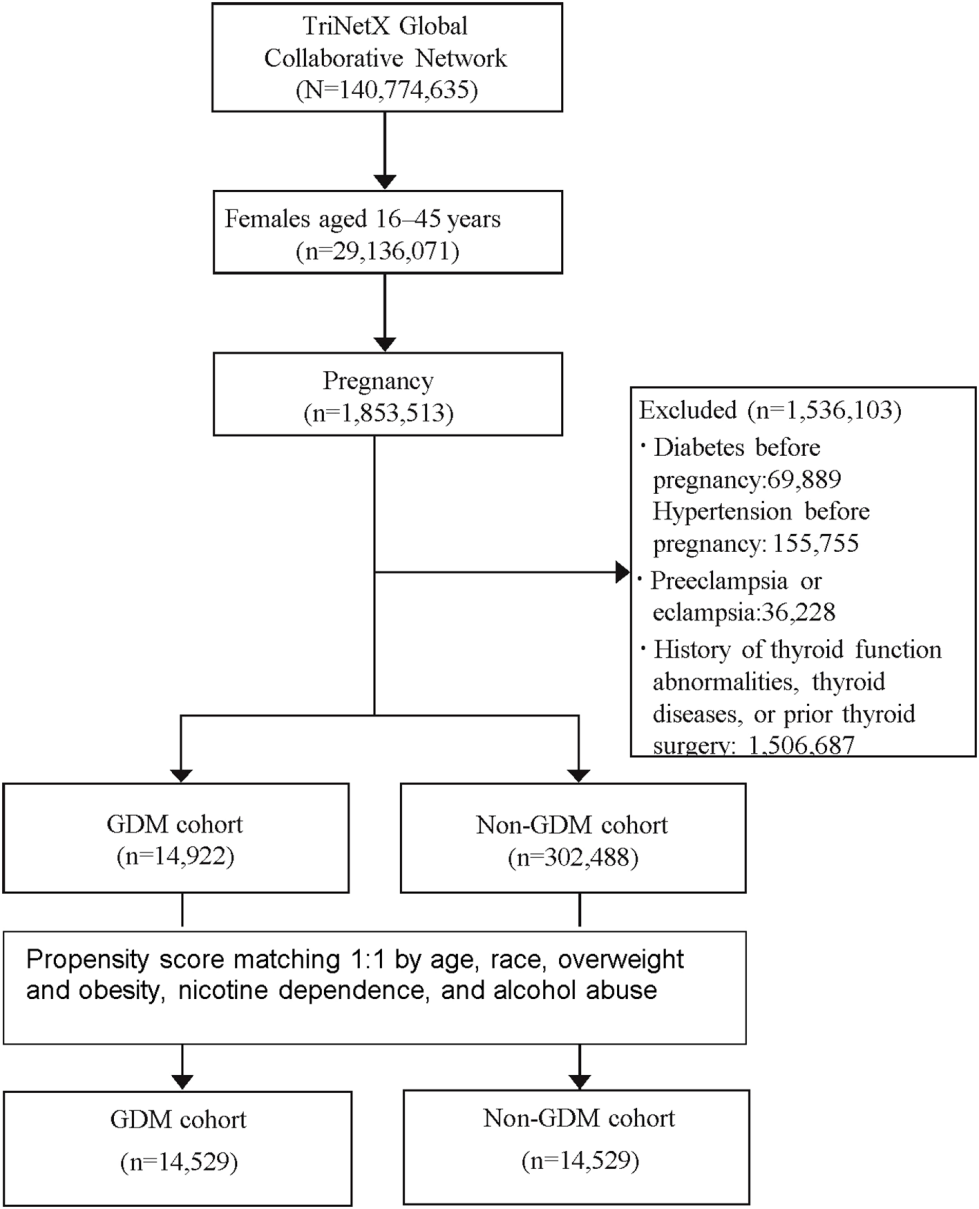
Study flowchart. GDM, Gestational Diabetes Mellitus.

The study aimed to evaluate the long-term risk of developing various thyroid-related disorders, including hyperthyroidism, hypothyroidism, toxic and non-toxic goiter, thyroiditis (acute, subacute, and Hashimoto’s), benign thyroid neoplasm, and thyroid cancer. A detailed list of these conditions and their corresponding diagnostic codes is provided in **Supplementary Table S1**. The study design and patient selection algorithms are illustrated in **Figure 1**.

Acute thyroiditis, subacute thyroiditis, and postpartum thyroiditis were initially included as predefined outcomes. However, each of these conditions had only 10 identified cases during follow-up, which likely reflects the tendency of clinicians to classify them under the broader diagnostic category of “thyroiditis” without further specification. Consequently, these subtypes were excluded from subgroup analyses due to insufficient case numbers. Similarly, benign neoplasm of the thyroid gland, which also had only 10 identified cases, was excluded from subgroup analysis despite being retained in the overall outcome analysis.

### Statistical analysis

The study population was determined based on all eligible patients within the database who met the predefined inclusion and exclusion criteria between January 1, 2001, and December 31, 2015. Therefore, no *a priori* sample size calculation was performed; instead, the entire eligible population was included to maximize statistical power and generalizability.

Propensity Score Matching (PSM) was employed using the integrated TriNetX platform to generate 1:1 matched cohorts with comparable baseline characteristics. Matching was restricted to covariates without missing data, and separate PSM procedures were conducted for stratified analyses using a limited set of covariates. Key matching variables included age, race, overweight/obesity, nicotine dependence, and alcohol abuse. The PSM was performed using a greedy nearest neighbor algorithm with a 0.1 caliper based on pooled standard deviations. Covariate balance was assessed using standardized mean differences (SMDs), with an SMD of <0.1 indicating good balance.

After matching, hazard ratios (HRs) with 95% confidence intervals (CIs) were calculated using chi-square tests to evaluate associations between exposure and outcomes. Kaplan–Meier curves and log-rank tests were used to assess cumulative incidence, with HRs provided for group comparisons. Statistical significance was defined as a two-sided p-value < 0.05. All analyses were conducted exclusively through the TriNetX online platform.

## Result

A total of 317,410 patients met the study’s inclusion criteria, comprising 14,922 patients in the GDM cohort and 302,488 in the non-GDM cohort. Following PSM, 14,529 patients were included in each cohort, ensuring a well-balanced study population for comparison. The average age at the index was 29 years, with a diverse ethnic distribution: 44.8% White, 13.7% Black or African American, 13.8% Asian, 10.6% other races, and 13.1% unknown race. In both cohorts, approximately 20% of patients were classified as overweight or obese, 7% had nicotine dependence, and 1% had alcohol abuse (**Table 1**).

**Table 1:** Baseline characteristics of study population.

Variable	Before PSM		SMD	After PSM		SMD
	GDM	Non- GDM		GDM	Non- GDM	
	(n= 14,922)	(n= 302,488)		(n=14,529)	(n=14,529)	
Age at Index (mean ± SD)	29.5 ± 4.7	26.0 ± 4.8	0.728	29.3 ± 4.6	28.9 ± 4.2	0.091
Race, n (%)						
White	6,598 (44.6)	136,107 (46.0)	0.028	6,506 (44.8)	6,491 (44.7)	0.002
Black or African American	2,010 (13.6)	53,851 (18.2)	0.126	1,986 (13.7)	2,064 (14.2)	0.016
Asian	2,034 (13.7)	30,944 (10.5)	0.101	1,999 (13.8)	1,873 (12.9)	0.026
Other Race	1,592 (10.8)	22,747 (7.7)	0.107	1,546 (10.6)	1,519 (10.5)	0.006
Unknown Race	1,933 (13.1)	39,764 (13.4)	0.011	1,898 (13.1)	2,007 (13.8)	0.022
Comorbidities, n (%)						
Overweight and Obesity	3,182 (21.5)	13,601 (4.6)	0.519	2,917 (20.1)	3,326 (22.9)	0.069
Nicotine dependence	1,089 (7.4)	11,105 (3.8)	0.158	1,015 (7.0)	1,239 (8.5)	0.058
Alcohol abuse	158 (0.048)	1,856 (0.6)	0.048	148 (1.0)	195 (1.3)	0.03

GDM, Gestational Diabetes Mellitus; PSM, Propensity Score Matching; SMD, Standardized Mean Difference.

Our study revealed a significant increase in the risk of thyroid function abnormalities and thyroid disease in the GDM cohort compared to the non-GDM cohort. Specifically, the risks of both hyperthyroidism and hypothyroidism were elevated, with hazard ratios (HR) of 1.60 (95% CI: 1.30–1.99) and 1.33 (95% CI: 1.17–1.51), respectively. Additionally, the GDM cohort showed a significantly higher risk for specific thyroid diseases, including toxic goiter (HR 1.70, 95% CI: 1.19–2.44), non-toxic thyroid goiter (HR 1.26, 95% CI: 1.10–1.45), thyroiditis (HR 1.55, 95% CI: 1.21–2.00), and Hashimoto’s thyroiditis (HR 1.37, 95% CI: 1.02–1.83). However, no significant difference was observed between the two cohorts in the incidence of benign thyroid neoplasms or thyroid cancer (**Table 2**).

**Table 2:** HRs and 95% CIs for the risk of thryoid disease according to the status of gestational diabetes mellitus (n=14,529).

Outcome	Patients with outcome (n)		HR (95% CI)
	GDM	Non-GDM	
Thyroid function			
Hyperthyroidism	205	145	1.60 (1.30,1.99)
Hypothyroidism	518	442	1.33 (1.17,1.51)
Thyroid disease			
Toxic Goiter	74	50	1.70 (1.19,2.44)
Non-toxic Goiter	423	383	1.26 (1.10,1.45)
Thyroiditis	412	104	1.55 (1.21,2.00)
Acute thyroiditis	10	10	1.13 (0.16,8.05)
Subacute thyroiditis	10	10	1.71 (0.48,6.06)
Hashimoto’s thyroiditis	108	79	1.37 (1.02,1.83)
Postpartum thyroiditis	10	15	0.55 (0.23,1.30)
Malignant neoplasm of thyroid gland	45	37	1.39 (0.83,2.31)
Benign neoplasm of thyroid gland	12	10	1.74 (0.71,4.25)

HR, hazard ratio; CI, confidence interval; GDM, Gestational Diabetes Mellitus.

Subgroup analysis by age showed no significant differences in the risk of thyroid function abnormalities or thyroid diseases between the GDM and non-GDM groups among individuals aged 16–34 years. However, among those aged 35–45 years, the risk of both hyperthyroidism and hypothyroidism increased by approximately 1.5-fold. This age group also demonstrated significantly higher risks of specific thyroid disorders, with hazard ratios of 1.75 (95% CI: 1.32–2.31) for thyroiditis, 1.67 (95% CI: 1.21–2.30) for Hashimoto’s thyroiditis, 1.87 (95% CI: 1.25–2.79) for toxic goiter, and 1.37 (95% CI: 1.18–1.59) for non-toxic thyroid goiter (**Table 3**).

**Table 3:** HRs and 95% CIs for the risk of thyroid disease according to the status of age and gestational diabetes mellitus.

Outcome	Young age patients with outcome (n=1,800)		HR (95% CI)	Advanced age patients with outcome (n=10,753)		HR (95% CI)
	GDM	Non-GDM		GDM	Non-GDM	
Thyroid function						
Hyperthyroidism	21	25	0.921 (0.515,1.647)	180	128	1.53 (1.22,1.92)
Hypothyroidism	56	49	1.247 (0.849,1.830)	443	333	1.45 (1.26,1.67)
Thyroid disease						
Toxic Goiter	10	10	0.866 (0.322,2.328)	65	38	1.87 (1.25,2.79)
Non-toxic Goiter	27	36	0.816 (0.495,1.344)	387	310	1.37 (1.18,1.59)
Thyroiditis	14	17	0.908 (0.447,1.844)	126	79	1.75 (1.32,2.31)
Hashimoto’s thyroiditis	12	11	1.237 (0.545,2.807)	94	62	1.67 (1.21,2.30)
Thyroid cancer	10	0	- (-,-)	32	33	1.05 (0.65,1.71)

HR, hazard ratio; CI, confidence interval; GDM, Gestational Diabetes Mellitus.

The study revealed that thyroid-related outcomes varied depending on BMI. In this research, BMI was assessed based on measurements taken within six months prior to pregnancy. Among patients with a BMI greater than 25, the risks of hyperthyroidism, hypothyroidism, thyroiditis, Hashimoto’s thyroiditis, non-toxic thyroid goiter, and thyroid cancer were significantly increased, with hazard ratios (HR) of 1.53 (1.18–1.98), 1.28 (1.10–1.50), 1.44 (1.06–1.96), 1.45 (1.02–2.05), 1.23 (1.04–1.45), and 2.40 (1.20–4.79), respectively. However, in patients within the normal BMI range (18.5–24.9), only non-toxic thyroid goiter showed an increased risk, approximately 1.3 times higher. (**Table 4**).

**Table 4:** HRs and 95% CIs for the risk of thyroid disease according to the status of BMI and gestational diabetes mellitus.

Outcome	BMI>25 patients with outcome (n=8,494)		HR (95% CI)	BMI 18.5-24.9 patients with outcome (n=3,223)		HR (95% CI)
	GDM	Non-GDM		GDM	Non-GDM	
Thyroid function						
Hyperthyroidism	137	102	1.53 (1.18,1.98)	46	39	1.41 (0.92,2.16)
Hypothyroidism	353	314	1.28 (1.10,1.50)	117	115	1.21 (0.93,1.56)
Thyroid disease						
Toxic Goiter	48	40	1.38 (0.91,2.10)	12	10	1.63 (0.68,3.87)
Non-toxic Goiter	284	266	1.23 (1.04,1.45)	110	99	1.33 (1.01,1.75)
Thyroiditis	94	74	1.44 (1.06,1.96)	34	31	1.29 (0.79,2.11)
Hashimoto’s thyroiditis	72	57	1.45 (1.02,2.05)	27	26	1.25 (0.73,2.14)
Thyroid cancer	25	12	2.40 (1.20,4.79)	10	10	2.06 (0.69,6.14)

HR, hazard ratio; CI, confidence interval; GDM, Gestational Diabetes Mellitus.

Subgroup analysis by medication status revealed elevated risks of thyroid dysfunction and disease in both GDM groups compared to non-GDM individuals (**Table 5**). Among patients with GDM receiving medication, the risks of hyperthyroidism (HR: 1.90; 95% CI: 1.19–3.03) and hypothyroidism (HR: 1.62; 95% CI: 1.23–2.15) were significantly increased. Similar trends were observed in the non-medicated GDM group, with HRs of 1.59 (95% CI: 1.26–2.01) and 1.33 (95% CI: 1.15–1.54), respectively. For thyroid diseases, the medicated group showed higher risks of toxic goiter (HR: 3.40; 95% CI: 1.40–8.24) and non-toxic goiter (HR: 1.67; 95% CI: 1.24–2.27), while the non-medicated group had modestly elevated risks (toxic goiter: HR: 1.47; 95% CI: 1.00–2.15; non-toxic goiter: HR: 1.22; 95% CI: 1.05–1.42). The non-medicated group also had significantly increased risks of thyroiditis (HR: 1.56; 95% CI: 1.18–2.06), Hashimoto’s thyroiditis (HR: 1.63; 95% CI: 1.18–2.25), and thyroid cancer (HR: 2.44; 95% CI: 1.32–4.51). In contrast, these associations were not statistically significant in the medicated group.

**Table 5:** HRs and 95% CIs for the risk of thyroid disease according to the status of medication and gestational diabetes mellitus.

Outcome	Patients with outcome (n=2,897)		HR (95% CI)	Patients with outcome (n=11,446)		HR (95% CI)
	GDM with medication	Non-GDM		GDM without mediation	Non-GDM	
Thyroid function						
Hyperthyroidism	45	29	1.90 (1.19,3.03)	168	120	1.59 (1.26,2.01)
Hypothyroidism	117	87	1.62 (1.23,2.15)	408	345	1.33 (1.15,1.54)
Thyroid disease						
Toxic Goiter	18	10	3.40 (1.40,8.24)	60	47	1.47 (1.00,2.15)
Non-toxic Goiter	100	73	1.67 (1.24,2.27)	349	349	1.22 (1.05,1.42)
Thyroiditis	27	25	1.34 (0.77,2.32)	119	87	1.56 (1.18,2.06)
Hashimoto’s thyroiditis	22	18	1.55 (0.83,2.91)	89	63	1.63 (1.18,2.25)
Thyroid cancer	10	10	1.23 (0.36,4.26)	32	15	2.44 (1.32,4.51)

HR, hazard ratio; CI, confidence interval; GDM, Gestational Diabetes Mellitus.

## Discussion

In this study involving 317,410 pregnant women and 14,529 matched controls, we identified a significant association between GDM and an increased risk of thyroid function abnormalities and thyroid diseases. The risks of both hyperthyroidism and hypothyroidism were elevated, along with a higher incidence of thyroiditis, Hashimoto’s thyroiditis, goiter with thyrotoxicosis, and non-toxic thyroid goiter in the GDM cohort. These risks were particularly pronounced in individuals aged 35–45 and those with a BMI over 25. Additionally, the severity of GDM, whether managed with or without medication, did not modify the increased risk of thyroid dysfunction.

GDM is typically defined as glucose intolerance that is usually diagnosed at 24–28 weeks’ gestation. In patients with GDM, factors or conditions that lead to maternal pancreatic β-cell dysfunction or delayed β-cell response result in reduced insulin secretion, ultimately causing maternal hyperglycemia (3). Elevated circulating levels of human chorionic gonadotropin (hCG) and its thyrotropic effects significantly alter pre-pregnancy thyroid hormone homeostasis. Additionally, increased placental deiodinase activity and higher serum concentrations of thyroxine-binding globulin (TBG) have been observed. Due to hCG stimulation, an increase in thyroid volume is noted throughout pregnancy (12). Thus, pregnancy not only affects blood glucose levels but also has a profound impact on thyroid morphology and function.

A meta-analysis has shown that hypothyroxinemia, overt hypothyroidism, subclinical hypothyroidism, overt hyperthyroidism, and the presence of thyroid antibodies are all significantly associated with an increased risk of GDM (13). However, a retrospective study from the opposite perspective found that patients with GDM had higher TSH levels and a higher prevalence of TPO and TG antibody positivity compared to those without GDM, suggesting a potentially bidirectional relationship between GDM and thyroid function or disease (4). While previous studies have primarily focused on the association between thyroid function—either pre-pregnancy or during pregnancy—and GDM, no research to date has examined the long-term effects of GDM on subsequent thyroid function or thyroid disorders. This study is the first to explore this relationship and has identified a significant association between GDM and future thyroid dysfunction and disease.

Regarding hyperthyroidism and hypothyroidism, the association between GDM and hypothyroidism appears to be more pronounced. A meta-analysis found that subclinical hypothyroidism with positive thyroid autoantibodies significantly increased the risk of GDM (OR 3.22, 95% CI: 1.72–6.03) (14). A cross-sectional study investigating the prevalence and risk of thyroid dysfunction in pregnant women with DM or GDM found that 10.85% had subclinical hypothyroidism, and 18.69% had hypothyroidism. In contrast, only 2.02% had subclinical hyperthyroidism, and 1.77% had hyperthyroidism (15). Consistently, our long-term follow-up showed a higher incidence of hypothyroidism in the GDM cohort. However, the risk of hyperthyroidism was also significantly elevated, with hazard ratios of 1.33 for hypothyroidism and 1.60 for hyperthyroidism. Importantly, our study further examined the long-term risk of specific thyroid disorders—including toxic goiter, non-toxic goiter, thyroiditis, and Hashimoto’s thyroiditis—and identified significant associations with GDM. To our knowledge, no previous research has comprehensively reported these disease-specific risks in this population.

Two studies have indicated that the prevalence of hypothyroidism increases with age (16, 17). Although data from the general population suggest that hypothyroidism should be more common in older pregnant women, a cohort study specifically evaluating this hypothesis failed to show any increase in the prevalence of hypothyroidism among women over 30 compared to younger women (18). The impact of maternal age on the development of thyroid disease in GDM patients has not been thoroughly explored. In our study, we found that individuals with GDM who were of advanced maternal age had a significantly higher likelihood of developing thyroid dysfunction or disease later in life compared to the non-GDM group. However, no difference was observed in younger individuals between the GDM and non-GDM groups.

Previous studies have suggested a potential correlation between BMI and thyroid function (19). Among individuals with severe obesity, the prevalence of overt and subclinical hypothyroidism was significantly higher, reaching 19.5% (20). Another study also showed that obese patients had lower free T3 and free T4 levels, a higher prevalence of hypothyroidism, and an increased occurrence of antithyroid antibodies (21). One meta-analysis by Rong-hua Song et al. further demonstrated that obesity was significantly associated with hypothyroidism, Hashimoto’s thyroiditis, and thyroid peroxidase antibodies, implying that prevention of obesity is crucial for reducing the burden of thyroid disorders (22). In pregnant women, research has indicated that elevated BMI during pregnancy is associated with an increased risk of developing hypothyroidism (15). In addition, a retrospective study confirmed that pre-pregnancy obesity may modify the association between maternal thyroid hormone sensitivity and the development of GDM, indicating a potential synergistic effect between impaired thyroid regulation and glucose metabolism in obese women (23). In our study, we found that among overweight and obese individuals, those with a history of GDM were more likely to develop hyperthyroidism or hypothyroidism over the long term compared to those without GDM. Interestingly, this association was not observed in participants with normal BMI, implying that high BMI may interact with GDM to increase thyroid disease risk through additive or synergistic mechanisms. While our findings highlight GDM as an important long-term risk factor, the contribution of elevated BMI—either as an independent risk factor or as an effect modifier—should not be overlooked.

When GDMis first diagnosed, blood sugar levels can be managed through dietary and lifestyle modifications. However, in up to 15-30% of GDM patients, despite recommendations for dietary and lifestyle changes, blood sugar control remains insufficient, necessitating medication therapy (24). In pregnancies affected by GDM, those requiring insulin treatment have a significantly higher risk of maternal and neonatal complications compared to those managed with diet alone (25). While it remains unclear whether the severity of GDM influences long-term thyroid function and disease development, our study found that regardless of whether GDM was mild and managed with diet alone or more severe and requiring medication, it still had a lasting impact on thyroid function and the occurrence of thyroid disease.

Our study has several limitations due to the constraints of the TriNetX database. We were only able to define gestational diabetes mellitus (GDM) based on ICD codes, which may introduce bias, as diagnostic criteria for GDM can vary slightly across different regions. Furthermore, we were unable to determine the number of pregnancies (parity) for each participant. Therefore, it remains unclear whether GDM represented a first-time diagnosis or a recurrent event in the same individual. Data on the gestational age at which GDM was diagnosed were not available, nor was information on pregnancy outcomes. Additionally, we could not assess weight changes during pregnancy or evaluate thyroid function both before and during pregnancy. These limitations may affect the comprehensiveness of our findings and highlight areas for further investigation in future studies.

In summary, women with GDM face an elevated risk of thyroid dysfunction and thyroid-related diseases in the long term. Clinicians and patients with a history of GDM should prioritize monitoring future thyroid health.

## Data Availability

The datasets presented in this study can be found in online repositories. The names of the repository/repositories and accession number(s) can be found below: The data that support the findings of this study are available in TriNetX official web site at https://trinetx.com/. These data were derived from the following resources available in the public domain.
