# Supplementary material for: Gestational diabetes: a contributor to long-term thyroid dysfunction and disease
Source: Front Endocrinol (Lausanne). 2025 Aug 29;16:1656498. doi: 10.3389/fendo.2025.1656498 (PMC12425773; doi:10.3389/fendo.2025.1656498)
Supplement: Supplementary file 1 [file Table1.docx]

Supplementary Table 1. Medical Codes of Cohort study

| Variable | Code(s) |
| --- | --- |
| Pregnancy | ICD-10-PCS 10 |
| Diabetes mellitus | ICD-10-CM E08-E13 |
| Hypertension | ICD-10-CM I10 |
| Gestational diabetes mellitus | ICD-10-CM O24.4 |
| Preeclampsia | ICD-10-CM O14.00, 014.10 |
| Eclampisa | ICD-10-CM O15 |
| Hyperthyroidism | ICD-10-CM E05 |
| Hypothyroidism | ICD-10-CM E03 |
| Toxic thyroid goiter | ICD-10-CM E05.0, E05.01, E05.2, |
| Non-toxic thyroid goiter | ICD-10-CM E04 |
| Thyroiditis | ICD-10-CM E06 |
| Hashimoto’s thyroiditis | ICD-10-CM E06.3 |
| Acute thyroiditis | ICD-10-CM E06.0 |
| Subacute thyroiditis | ICD-10-CM E06.1 |
| Postpartum thyroiditis | ICD-10-CM O90.5 |
| Iodine deficiency related thyroid disorder | ICD-10-CM E01 |
| Malignant neoplasm of thyroid gland | ICD-10-CM C73 |
| Benign neoplasm of thyroid gland | ICD-10-CM D34 |
| Surgical procedures of thyroid gland | CPT 1009025 |
| Overweight and obesity | ICD-10-CM E66 |
| Nicotine dependence | ICD-10-CM F17 |
| Alcohol abuse | ICD-10-CM F10.1 |
